# The upper temperature and hypoxia limits of Atlantic salmon (*Salmo salar*) depend greatly on the method utilized

**DOI:** 10.1242/jeb.246227

**Published:** 2023-09-26

**Authors:** Rebeccah M. Sandrelli, A. Kurt Gamperl

**Affiliations:** Department of Ocean Sciences, Memorial University of Newfoundland and Labrador, St John's, NL, Canada, A1C 5S7

**Keywords:** CT_max_, Thermal tolerance, Heart rate, Hypoxia tolerance, Data loggers, Bradycardia

## Abstract

In this study, Atlantic salmon were: (i) implanted with heart rate (*f*_H_) data storage tags (DSTs), pharmacologically stimulated to maximum *f*_H_, and warmed at 10°C h^−1^ (i.e. tested using a ‘rapid screening protocol’); (ii) fitted with Doppler^®^ flow probes, recovered in respirometers and given a critical thermal maximum (CT_max_) test at 2°C h^−1^; and (iii) implanted with *f*_H_ DSTs, recovered in a tank with conspecifics for 4 weeks, and had their CT_max_ determined at 2°C h^−1^. Fish in respirometers and those free-swimming were also exposed to a stepwise decrease in water oxygen level (100% to 30% air saturation) to determine the oxygen level at which bradycardia occurred. Resting *f*_H_ was much lower in free-swimming fish than in those in respirometers (∼49 versus 69 beats min^−1^) and this was reflected in their scope for *f*_H_ (∼104 versus 71 beats min^−1^) and CT_max_ (27.7 versus 25.9°C). Further, the Arrhenius breakpoint temperature and temperature at peak *f*_H_ for free-swimming fish were considerably greater than for those tested in the respirometers and given a rapid screening protocol (18.4, 18.1 and 14.6°C; and 26.5, 23.2 and 20.2°C, respectively). Finally, the oxygen level at which bradycardia occurred was significantly higher in free-swimming salmon than in those in respirometers (∼62% versus 53% air saturation). These results: highlight the limitations of some lab-based methods of determining *f*_H_ parameters and thermal tolerance in fishes; and suggest that scope for *f*_H_ may be a more reliable and predictive measure of a fish's upper thermal tolerance than their peak *f*_H_.

## Teaser

**Summary:** Heart rate responses to environmental challenges in free-swimming salmon are different from those measured using more invasive techniques. This has important implications for predicting the susceptibility of fishes to climate change.

## INTRODUCTION

Global sea surface temperatures are expected to rise by ∼1.5°C in the next two decades (IPCC, 2022), and this will have a significant impact on many marine ectotherms, including fishes (Gamperl et al., 2020; Genin et al., 2020; Laufkötter et al., 2020; Pinsky et al., 2019; Reid et al., 2019; Viglione, 2021). In addition, an increase in the frequency and severity of storms and weather events (Bender et al., 2010) will increase temperature variability (Frölicher et al., 2018; Solomon, 2007; Szekeres et al., 2016; Viglione, 2021), and these temperature fluctuations present challenges for both wild and farmed fish.

Temperature has been described as the ‘abiotic master factor’ for fishes (Brett, 1971) as it controls/limits behaviour and physiological functions (Fry, 1947). Given that fish are ectotherms, and that their body temperature is largely dependent on that of the water they live in, changes in temperature impact many aspects of their biology, health and welfare (Alfonso et al., 2021; Brett, 1971, 1979; Gamperl et al., 2020; Oppedal et al., 2011; Pörtner and Farrell, 2008; Tromp et al., 2018; Wade et al., 2019). Fish have a preferred thermal range at which growth and performance are maximum, and large deviations from this range have detrimental impacts including decreased appetite (and thereby growth), reproductive fitness, increased stress, and temperature-related mortality (Alfonso et al., 2021; Burke et al., 2020; Dahlke et al., 2020; Gamperl et al., 2020; Reid et al., 2019).

**List of abbreviations**

ABT: Arrhenius breakpoint temperature
CT_max_: critical thermal maximum
DST: data storage tag
ECG: electrocardiogram
*f*_H_: heart rate
*f*_H,ABT_: heart rate at Arrhenius breakpoint temperature
*f*_H,crit_: oxygen level at the onset of bradycardia
*f*_H,max_: maximum heart rate
*f*_H,peak_: maximum heart rate during thermal challenge
*f*_H,scope_: heart rate range from 10°C to highest (peak) value
LOE: loss of equilibrium
OCLTT: oxygen- and capacity-limited thermal tolerance
*Q*_10_: temperature coefficient
*Q*_10,preABT_: temperature coefficient from 10°C up to the Arrhenius breakpoint temperature
RVM: relative ventricular mass
*T*_arr_: temperature at which heart rate arrhythmias begin
*T_f_*_H,peak_: temperature of peak heart rate during a thermal challenge
*T*_opt_: optimal temperature
TMS: tricaine methanesulfonate

Limitations in performance beyond a species-specific thermal optimum (*T*_opt_) have been linked to constraints in the capacity of aquatic organisms to meet their oxygen demands, as depicted in the widely recognized, but also controversial (Clark et al., 2013a,b; Jutfelt et al., 2014; Lefevre, 2016; Norin et al., 2014), oxygen- and capacity-limited thermal tolerance (OCLTT) theory originally described by Pörtner (Pörtner, 2002, 2010; Pörtner et al., 2017) which is based on Fry (1947). With regards to meeting the demands of increased temperature and determining a fish's upper thermal tolerance, an increase in heart rate (*f*_H_) plays a key role in increasing oxygen delivery to the tissues (Farrell et al., 2009; Wang and Overgaard, 2007) as: increases in cardiac output (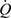; the amount of blood pumped by the heart per minute) with temperature are achieved solely by increases in *f*_H_ [i.e. stroke volume (*S*_V_), the amount of blood pumped per heart beat, is largely unchanged; Clark et al., 2008; Farrell and Smith, 2017; Gollock et al., 2006; Mendonça and Gamperl, 2010; Steinhausen et al., 2008; Stevens et al., 1972)]; and recent studies suggest that increases in *f*_H_ are critical to the survival of fish at high temperatures when water oxygen levels are decreased (i.e. when experiencing hypoxia) (Gamperl et al., 2021; Leeuwis et al., 2021). Indeed, it has been suggested that cardiac (*f*_H_) failure is functionally, and more ecologically, relevant than the temperature at which the fish loses equilibrium (i.e. reaches its critical thermal maximum, CT_max_) (Sidhu et al., 2014).

Given concerns about the effects of increasing ocean temperatures and heat waves on fish populations (Lefevre et al., 2021; Little et al., 2020; Pörtner and Knust, 2007; Pörtner et al., 2017; Sinclair et al., 2016; van der Walt et al., 2021), the scientific community has been determining the upper thermal tolerance of numerous fish species, and examining how this parameter relates to oxygen consumption and cardiac function (Anttila et al., 2014; Casselman et al., 2012; Hansen et al., 2017; Leeuwis et al., 2019; Mignucci et al., 2021; Motyka et al., 2017; Muller et al., 2020). The most widely used method to determine the acute upper thermal tolerance of fishes is the CT_max_ test (Becker and Genoway, 1979). This test/protocol involves increasing water temperature at a constant rate (°C h^−1^) from the fish's acclimation temperature until loss of equilibrium (LOE), defined as the loss of a fish's ability to maintain normal dorsal–ventral orientation (Brauner and Richards, 2020). The rate of temperature increase varies greatly in the literature, with values ranging from 18°C h^−1^ (Anttila et al., 2013; Åsheim et al., 2020; Becker and Genoway, 1979; Gallant et al., 2017) to ∼1–2°C h^−1^ (Blasco et al., 2020; Motyka et al., 2017; Leeuwis et al., 2019; Penney et al., 2014; Zanuzzo et al., 2019); the latter is the maximum that would be considered ecologically relevant under most conditions (Caissie et al., 2012; Desforges et al., 2023; Richards, 2011; Rodnick et al., 2004). Such tests have often been performed in respirometers so that oxygen consumption can be measured, and heart function can be monitored by implanting flow probes around the ventral aorta (Gamperl et al., 2011; Gollock et al., 2006; Keen and Gamperl, 2012; Mendonça and Gamperl, 2010). However, more recently, a ‘rapid screening protocol’ originally developed by Casselman et al. (2012) has been increasingly used to determine the thermal tolerance of fishes. In this protocol, fish are anaesthetized and placed supine in a water bath, injected with atropine (to block cholinergic tone on the heart) and isoproterenol (to ensure maximum adrenergic cardiac stimulation), and *f*_H_ is measured using subdermal electrocardiogram (ECG) electrodes as water temperature is increased rapidly (at 10–18°C h^−1^). However, there are concerns about how accurately *f*_H_–temperature relationships and indices used to estimate a species' thermal tolerance (e.g. Arrhenius breakpoint temperature, ABT; temperature at peak heart rate, *f*_H,peak_; temperature of cardiac arrhythmias, *T*_arr_) under these highly controlled/manipulated and unnatural conditions reflect those in free-swimming fishes (Motyka et al., 2017; also see references below).

Data storage tags (DSTs; also called data loggers) have recently been used to determine breakpoints in cardiac function (i.e. in *f*_H_) in anaesthetized Roman sea bream (*Chrysoblephus laticeps*; Skeeles et al., 2020) and white sea bream (*Diplodus capansis*; van der Walt et al., 2021) following the protocol developed by Casselman et al. (2012) and the authors acknowledge that there are limitations in using such manipulated conditions for estimating cardiac parameters. Further, Mignucci et al. (2021) recently compared the *f*_H_ of gilthead sea bream (*Sparus aurata*) that were implanted with DSTs while free-swimming in a tank versus recovered in a respirometer. These authors reported that fish in the respirometers had a higher resting *f*_H_ and suggested that biologging provides more reliable insights into the cardiac and behavioural responses of fish to environmental stressors. Clearly, more research needs to be conducted in this area so that we can understand the constraints of highly manipulated measurement conditions on fish cardiac function, and how they relate to measures of cardiac function (and thus thermal tolerance) in various species under ‘real world’ conditions. Such information is critical to ensuring that conservation and management policies implemented to protect fish species in the wild, or strategies to safeguard fish held in aquaculture operations, are based on accurate and reliable information about their thermal biology.

Therefore, the objective of this study was to compare measures of upper thermal (CT_max_) and hypoxia (the oxygen level at which bradycardia is initiated) tolerance determined using *f*_H_ DSTs in free-swimming fish (after 4 weeks of post-surgical recovery) with that of fish: (i) exposed to the rapid screening protocol of Casselman et al. (2012); and (ii) fitted with blood flow probes in a respirometer. These experiments were performed on Atlantic salmon because it is a eurythermal fish whose environmental tolerances have been reported in several studies (e.g. Anttila et al., 2013, 2014; Gallant et al., 2017; Hvas et al., 2017; Leeuwis et al., 2019; Penney et al., 2014), global populations of this species are declining (Dadswell et al., 2021; Chaput, 2012; Nicola et al., 2018; Mills et al., 2013), and it is an important aquaculture species that is experiencing elevated temperatures and hypoxic conditions at cage-sites in several regions (Burke et al., 2020; Burt et al., 2012; Gamperl et al., 2021; Oldham et al., 2017; Oppedal et al., 2011; Stehfest et al., 2017).

## MATERIALS AND METHODS

This research was approved by the Institutional Animal Care Committee of Memorial University of Newfoundland and Labrador (protocol ^#^21-05-KG) and performed in accordance with the Canadian Council on Animal Care Guidelines on the ‘Care and Use of Fish in Research, Teaching and Testing’ (https://ccac.ca/Documents/Standards/Guidelines/Fish.pdf).

### Fish husbandry

The Atlantic salmon (*Salmo salar*) used in these studies were age ≥1 year, of Saint John River origin, and originally supplied by Cooke Aquaculture Inc. (Ormocto, NB, Canada) as pre-smolts. These fish were smolted at the Dr Joe Brown Aquatic Research Building (Ocean Sciences Centre, Memorial University of Newfoundland and Labrador), and held there for ∼9 months, prior to being moved to the Laboratory for Atlantic Salmon and Climate Change Research (LASCCR, Ocean Sciences Centre). At the LASCCR, the fish were held in 2.2 m^3^ tanks supplied with seawater at 10°C and at ≥100% air saturation and exposed to a 14 h light:10 h dark photoperiod. During this period, they were fed 4 mm Signature Salmon Ration-PW (Northeast Nutrition Inc., Truro, NS, Canada) at a ration of 1% body mass (*M*_b_) day^−1^. In all experiments mass (g), fork length (cm) and ventricular mass (g) were recorded. The fish used in these experiments averaged 838.2±13.4 g in mass and 41.6±0.3 cm in length (means±s.e.m.).

### Experimental design

#### Group 1: rapid screening using anaesthetized fish

##### Surgery

Prior to implantation, micro-HRT DSTs (8.3 mm×25.4 mm, 3.3 g; Star Oddi, Garðabær, Iceland) were inserted into Star Oddi's communication interface (COM-BOX), and Mercury (v.6.02) software running on a computer was used to program them. The start date and time, sampling intervals and frequencies were all set using this software. The DSTs were set to record *f*_H_ (at 100 Hz for 6 s) and temperature (5–45°C; ±0.2°C) every 15 s for 4 recordings, followed by 1 recording where the ECG was saved, and this continued for the duration of the experiment. Raw ECGs were saved to validate the data collected (i.e. determine R–R intervals and *f*_H_).

Each fish was netted from their tank and anaesthetized in oxygenated seawater containing tricaine methanesulfonate (Syncaine TMS; 0.2 g l^−1^; Syndel Laboratories Canada, Nanaimo, BC, Canada) until ventilatory movements ceased. The fish were then placed supine on a wetted surgical sponge, and their gills were continuously supplied with ∼10°C oxygenated seawater containing a maintenance dose of TMS (0.05 g l^−1^). A small mid-ventral incision (1.1±0.03 cm), at the posterior limit of the base of the pectoral fins, was made in the fish's body wall using a scalpel. A micro-HRT DST was then inserted into the abdominal cavity (blunt-end first) towards the posterior of the fish, and then pulled forward using a suture (2-0) pre-tied at the middle of the DST to within 0.5 cm of the pericardium. A ½ circle, 28 mm, cutting edge needle (SE-MH 28, Mani Surgical Needles, Utsunomiya, Japan) was then used to pass the suture through the body wall and to start to close the incision. Finally, the incision was closed using 3-0 silk sutures: 2–4 interrupted stitches depending on incision length. Vaseline was applied to the incision to prevent water from entering the wound. DST implantation took approximately 5 min; thereafter, the fish was placed supine on a V-board and moved to a shallow water table (50 l) filled with 10°C oxygenated seawater containing a maintenance dose of TMS (0.05 g l^−1^). The fish were completely submerged in the seawater, and aerated seawater from the bath was constantly pumped over the fish's gills. After *f*_H_ and temperature data had been recorded for 1 h, the fish were given sequential injections of atropine sulfate (1.2 mg kg^−1^) and isoproterenol (4 µg kg^−1^) (Sigma-Aldrich Canada Co., Oakville, ON, Canada) via the caudal blood vessels to block vagal tone and to maximally stimulate cardiac adrenergic β-adrenoreceptors, respectively (Casselman et al., 2012). Both drugs were dissolved in a 0.9% sodium chloride (NaCl) solution, and frozen at −80°C, prior to use.

##### Temperature challenge

Thirty minutes following the drug injections, water temperature was increased from the fish's acclimation temperature (10°C) to 28°C at 10°C h^−1^ (Casselman et al., 2012) using a circulating water bath connected to a titanium coil submerged in the seawater. Water temperature was recorded using a PT 100 probe (−30–150°C; resolution: 0.02°C, accuracy: ±0.5°C) connected to a Firesting O_2_ meter and a computer running Pyro Oxygen Logger software (PyroScience GmbH, Aachen, Germany). *f*_H_ and the fish's internal temperature were measured using the DSTs. In addition, in a subset of experiments (*n*=3), a calibrated thermocouple (Model: HHC201, Type K Thermocouple, −100–1372°C, accuracy: 0.1%; Omega Engineering Inc., Norwalk, CT, USA) was placed directly under the liver for the duration of the experiment to validate the DST's values of internal body temperature. Thereafter, the fish were euthanized in 0.4 g l^−1^ TMS. Following euthanasia, the heart was removed from the fish, and the bulbous arteriosus and atrium were separated from the ventricle. The ventricle was then blotted dry on Kimwipes and weighed. Finally, the DST was retrieved, and the data downloaded.

##### Hypoxia challenge

No hypoxia challenge was performed on this group. Atropine sulfate blocks vagal tone and, thus, the fish are unable to initiate bradycardia/slow their *f*_H_ when exposed to hypoxic water.

#### Group 2: traditional respirometry

##### Surgery

Each fish was netted from their tank and anaesthetized in oxygenated seawater containing TMS (0.2 g l^−1^) until ventilatory movements ceased. The fish were placed on a wet foam pad on a surgical table while their gills were irrigated continuously with oxygenated seawater containing a maintenance dose of TMS (0.1 g l^−1^). Then, the salmon were placed on their right side, and umbilical tape was passed under the gill arches and secured to the surgical table to allow access to the opercular cavity. A small puncture was then made in the skin just below the junction of the second and third gill arches with a pair of sharp-pointed forceps, and the ventral aorta was carefully located by expanding the hole using blunt dissection. Once identified, the ventral aorta was freed from the surrounding tissue using a pair of curved forceps without damaging the pericardium, and a Doppler^®^ flow probe (Model ES Cuff-type Transducer, 20 MHz, Iowa Doppler Products, Iowa City, IA, USA), 1.3 mm in diameter, was fitted around the ventral aorta. Finally, the flow probe lead was connected to a directional pulsed Doppler^®^ flow meter (Model 545C-4; Bioengineering, University of Iowa, Ames, IA, USA) interfaced with a MP100 data acquisition system connected to a laptop computer running Acknowledge (v.3.8.2, BioPac Systems Inc., Goleta, CA, USA) to ensure that the signal was of high quality, and the probe lead was secured to the fish at three locations using 3-0 silk suture: one suture immediately ventral to the pectoral fin, one just below the lateral line and one in front of the dorsal fin.

Upon completion of surgery, individual fish were placed in a ∼20 l cylindrical respirometer (20 cm diameter×54.6 cm length) submerged in a shallow (25 cm deep) water table containing fully aerated seawater at 10°C. The respirometer received a constant flow of water at a rate of 10 l min^−1^ from a submersible pump (model 1048; EHEIM GmbH & Co., Deizisau, Germany). Water in the experimental water table was supplied from a large (∼300 l) reservoir whose temperature was controlled by a custom-designed heater/chiller (Technical Services, Memorial University of Newfoundland and Labrador). The fish were allowed to recover/acclimate inside the respirometers for ∼20 h (i.e. until the first morning prior to the hypoxia challenge).

##### Hypoxia challenge

Approximately 20 h following surgery and acclimation to the respirometers, water oxygen levels were decreased step-wise every 60 min until the O_2_ level of 30% air saturation was reached (Fig. S1). Water air saturation was lowered by bubbling nitrogen gas into the seawater reservoir as controlled using a computer running WitroxCTRL software (Loligo Systems, Viborg, Denmark) that was interfaced with a fibre optic O_2_ meter and an O_2_ and temperature probe (Loligo Systems). This system controlled two solenoid valves which released air or nitrogen gas into the reservoir as required. Measurements of *f*_H_ were taken at every 5% decrease. *f*_H_ was recorded using the Doppler^®^ flow probe and recording system, as previously described, and *f*_H_ (beats min^−1^) was determined by averaging three sections of 15 consecutive systolic peaks.

After the measurements at 30% air saturation were taken, O_2_ in the respirometers was increased to 100% over ∼30 min. The fish were left undisturbed in the respirometers for ∼36 h at their acclimation temperature (10°C) to recover.

##### Temperature challenge

An acute upper thermal challenge to the fish's CT_max_ was used to determine the salmon's upper thermal tolerance. This is a standard protocol in the Gamperl lab (see Norin et al., 2019; Zanuzzo et al., 2019; Leeuwis et al., 2019; Motyka et al., 2017; Ern et al., 2016), in which water temperature is increased by 2°C h^−1^ until the fish loses equilibrium (LOE) (Fig. S1). *f*_H_ was recorded at each 1°C increment. The temperature at which LOE occurred was used as the CT_max_ value. Thereafter, the fish were euthanized with 0.4 g l^−1^ TMS, the heart was removed, and the bulbous arteriosus and atrium were separated from the ventricle. The ventricle was then blotted dry on Kimwipes and weighed. For the hypoxia experiment, *f*_H,crit_ was determined as the point at which bradycardia occurred, and ABT, *f*_H,ABT_, *Q*_10,preABT_, *f*_H,scope_ and *f*_H,peak_ were determined as described below.

#### Group 3: free-swimming fish with DSTs

##### Surgery

Prior to implantation, the DSTs (micro-HRT; Star Oddi) were set to record *f*_H_ (at 100 Hz for 6 s) and temperature (5–45°C; ±0.2°C) every 2 h for 24 h prior to the hypoxia challenge and between the hypoxia challenge and thermal challenge, and every 10 min during the hypoxia and thermal challenges. Raw ECGs were saved with every recording to determine R–R intervals (*f*_H_).

The micro-HRT tags were implanted in the fish as described above. The fish were then placed into a 2.2 m^3^ round tank with ∼40 conspecifics (stocking density 18 kg m^−3^), that was supplied with seawater at 10°C and with ≥100% air saturation in the LASCCR facility for 3 weeks. This duration of recovery was used as Zrini and Gamperl (2021) showed that this post-surgical period is required for resting *f*_H_ to return to stable levels. During this period, the fish were hand fed 1× daily at 1% *M*_b_ day^−1^ and maintained on a 14 h light:10 h dark photoperiod. Seven days prior to the hypoxia challenge, tagged fish (*n*=6 per experiment, see below) were moved into a 0.5 m^3^ tank and remained in this tank for a week before the hypoxia and thermal challenges.

##### Hypoxia challenge

The oxygen level was decreased by bubbling nitrogen in the header tank which supplied the tank with 5 l min^−1^ of seawater. A step-wise decrease in oxygen every 60 min was achieved, with steps of 100%, 80%, 70%, 60%, 50%, 40% and 30% air saturation as described above (Fig. S1). Temperature and oxygen were continuously monitored using a YSI 5500D MultiDO Optical Monitoring and Control Instrument (Yellow Springs Instruments, Yellow Springs, OH, USA), and a GoPro^®^ camera mounted above the tank was used to monitor the fish in real-time and record behaviour throughout the experiment. Following the last step, oxygen was increased over ∼30 min to ∼100% air saturation, and the fish were left undisturbed for 36 h.

##### Temperature challenge

A CT_max_ test was used to determine the fish's upper thermal tolerance. Water temperature was increased by 2°C h^−1^ by increasing temperature in the header tank that supplied the tank with seawater, while maintaining water oxygen levels at ∼100% air saturation (Fig. S1). As in the hypoxia experiment, a YSI 5500D MultiDO Optical Monitoring and Control Instrument (Yellow Springs Instruments) and a GoPro^®^ camera, were used to monitor the water and fish in real-time, respectively. The temperature at which LOE occurred was recorded as the CT_max_ value. Following LOE, the fish were euthanized with 0.4 g l^−1^ TMS, the heart was removed and the ventricle was weighed. Then, the DSTs were recovered. Two experiments were conducted, and all fish were exposed to the hypoxia challenge prior to the temperature challenge (*n*=6 per experiment, *n*=12 fish total).

The data from the DSTs were downloaded using the COM-BOX and analysed using Mercury software running on a computer. For the first experiment, *f*_H,crit_ was determined as the point at which bradycardia occurred, and ABT, *f*_H,ABT_, *Q*_10,preABT_, *f*_H,scope_ and *f*_H,peak_ were determined as described below.

### Data analysis

In these experiments, relative ventricular mass (RVM) was calculated as:
(1)

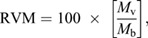
where *M*_v_ is ventricle mass and *M*_b_ is body mass (both measured in grams). The oxygen at which bradycardia occurred (*f*_H,crit_) was determined by plotting *f*_H_ against air saturation (%) to find the change in slope for each individual. This was determined for each individual using the segmented package (v.1.4-1) in R, which uses a piecewise regression to determine the breakpoint in the relationship. However, *f*_H,crit_ could not be determined for 2 fish tested in the respirometers (*n*=8). The normal exponential increase in *f*_H_ with temperature becomes discontinuous prior to arrhythmia (Casselman et al., 2012; Somero, 2011). The temperature at which this transition occurs is termed the ABT. ABT and the *f*_H_ at the ABT (*f*_H,ABT_) were determined for each fish by plotting the natural logarithm of *f*_H_ against the inverse of temperature (in Kelvin) and finding the point of change in slope using the segmented package (v.1.4-1) in R (Casselman et al., 2012) (e.g. Fig. S2). However, ABT could not be accurately determined for 3 fish tested in the respirometers and 5 fish free-swimming in the tank (*n*=7 for both groups). The *Q*_10_ for *f*_H_ was calculated for each individual across the temperature range from 10°C to a temperature just below the ABT (*Q*_10,preABT_) using the formula:
(2)

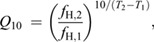
where *f*_H,1_ and *f*_H,2_ are the heart rate at the first (*T*_1_) and second (*T*_2_) temperatures, respectively. The scope for *f*_H_ (*f*_H,scope_) was calculated as the difference between *f*_H_ at 10°C and the highest recorded *f*_H_ (*f*_H,peak_), regardless of temperature. Graphs were created using Prism 9 (www.graphpad.com) and statistical analyses was completed using R (http://www.R-project.org/). Assumptions of normality and homogeneity of variance were performed by visual inspection of *Q*–*Q* plots and histograms of residuals. Resting (or ‘undisturbed’) values for *f*_H_ were compared between groups using a one-way ANOVA using values at the beginning of sedation or resting measurements (at 10°C and 100% air saturation). Differences in morphometric measurements (RVM, mass, length), CT_max_, ABT, *f*_H,ABT_, *Q*_10,preABT_, *f*_H,scope_ and *f*_H,peak_ were compared between the experimental groups using one-way ANOVAs (Table S1). Unpaired *t*-tests were used to compare CT_max_, *f*_H,crit_ and *f*_H_ at 100% and 30% air saturation for fish tested in the respirometers and free-swimming in the tank (Table S1). Linear mixed-effects models (LME) with temperature/oxygen and treatment (i.e. group) as fixed effects, an interaction term for the two, and fish as a random factor, were used to assess changes in *f*_H_. A linear regression was used to plot the relationship between *f*_H,scope_ and the temperature at *f*_H,peak_ (*T_f_*_H,peak_) for all groups. A linear regression was fitted to the *f*_H_ versus temperature relationship of free-swimming fish from 10 to 26°C during the CT_max_ test. The level of statistical significance used was *P*<0.05, and all values in the text, tables and figures are means±1 s.e.m.

## RESULTS

### Body and cardiac morphometrics

There were no differences in fish size (mass or length) between the experiments (*P*>0.05) (Table 1). However, the RVM of fish tested using traditional respirometry was significantly higher than that of fish assessed using the rapid screening protocol (0.090±0.004 versus 0.077±0.003, *P*<0.05) (Table 1). The RVM of free-swimming fish with implanted DSTs was not significantly different from that of the other treatments (*P>*0.05) (Table 1).

**Table 1.**
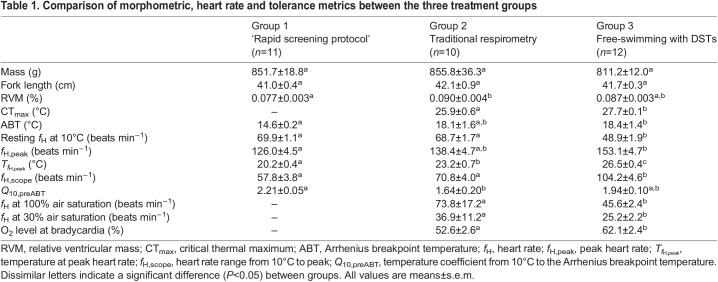
Comparison of morphometric, heart rate and tolerance metrics between the three treatment groups

### Resting *f*_H_

**Fig. 1.**
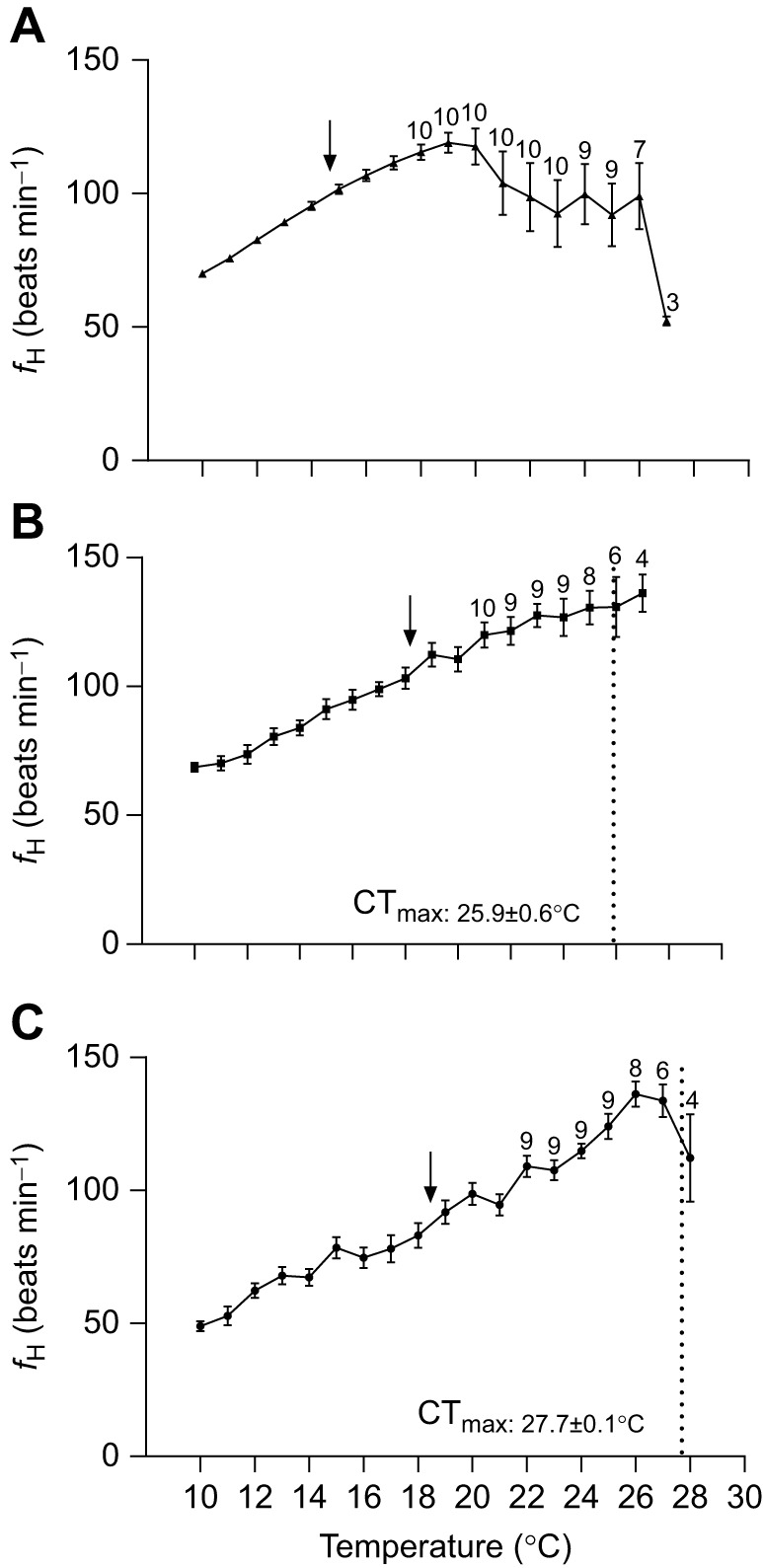
**Comparison of changes in the heart rate (*f*_H_) of Atlantic salmon during a temperature challenge.** *f*_H_ is shown for (A) fish implanted with data storage tags (DSTs) and given a ‘rapid screening protocol’ (*n*=11), (B) fish tested using traditional respirometry and implanted with Doppler^®^ flow probes (*n*=10) and (C) free-swimming fish held with conspecifics and implanted with DSTs (*n*=12). Numbers above the symbols indicate decreased sample size at a specific temperature. The average critical thermal maximum (CT_max_) value (mean±s.e.m.) is shown using a dotted line in B and C. Arrows indicate the Arrhenius breakpoint temperature (ABT). Temperature was increased at 10°C h^−1^ in A and at 2°C h^−1^ in B and C.

Resting *f*_H_ values at 10°C were significantly lower in the free-swimming fish with DSTs (48.9±1.9 beats min^−1^, *P<*0.05; Table 1, Fig. 1) than in fish assessed using the rapid screening protocol and traditional respirometry (69.9±1.1 and 68.7±1.7 beats min^−1^, respectively; Table 1, Fig. 1).

### Acute hypoxia challenge

**Fig. 2.**
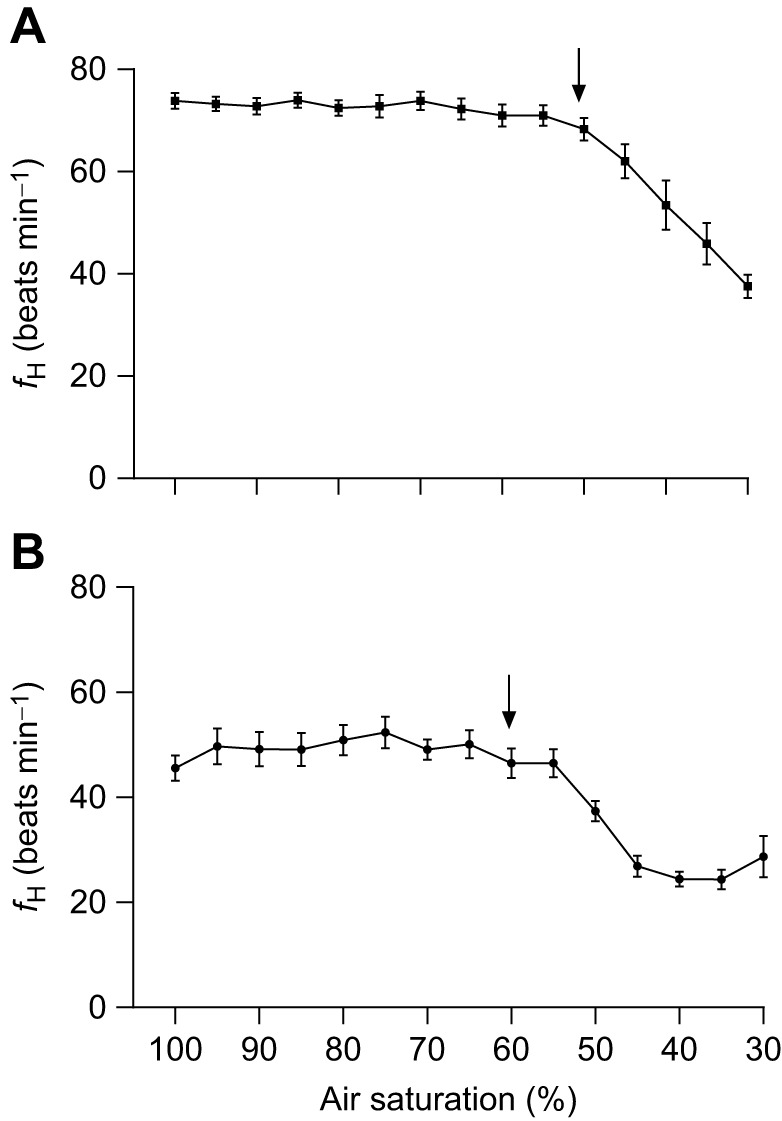
**Comparison of changes in *f*_H_ as water oxygen level was lowered from 100% to 30% air saturation.** Average *f*_H_ values at each 5% decrease in air saturation are plotted for (A) fish fitted with Doppler^®^ flow probes and tested using traditional respirometry (*n*=10) and (B) free-swimming fish in a tank with conspecifics and implanted with DSTs (*n*=12). The arrows indicate the average ABT as determined using individual fish.

The *f*_H_ values for free-swimming fish with DSTs and fish tested in the respirometers remained consistent with values at 100% air saturation until the initiation of bradycardia. The initiation of bradycardia (*f*_H,crit_) was significantly different between groups and occurred at 62.1±2.4% air saturation in free-swimming fish and 52.6±2.6% air saturation in fish tested in the respirometers (*P*<0.05) (Table 1, Fig. 2). The mean *f*_H_ in both groups was significantly lower by 45% air saturation than the initial *f*_H_ (at 100% air saturation). *f*_H_ for fish in the respirometers continued to decline linearly from the initiation of bradycardia until 30% air saturation whereas the *f*_H_ of free-swimming fish reached a plateau at ∼45% air saturation and remained between 24 and 28 beats min^−1^, ∼40% below initial values. Reduced swimming/movement of free-swimming fish was observed as O_2_ level was decreased and many (∼50% of fish) struggled to maintain equilibrium at 30% air saturation, whereas there were no observable changes in behaviour for fish tested in respirometers. At 30% air saturation, *f*_H_ was significantly lower in free-swimming fish (25.2±2.2 beats min^−1^) than in fish in the respirometers (36.9±11.2 beats min^−1^) (Table 1, Fig. 2). The average *f*_H_ decreased by ∼40% in the free-swimming fish and ∼50% for fish in respirometers (by ∼20 and 37 beats min^−1^, respectively) at 30% air saturation as compared with normoxic values.

### Temperature challenge

The ABT was significantly lower (14.6±0.2°C) using the rapid screening protocol than when fish were tested in the respirometers and in free-swimming fish (18.1±1.6 and 18.4±1.4°C, respectively) (Table 1). *f*_H,peak_ was also significantly lower using the rapid screening protocol compared with that of free-swimming fish (126.0±4.5 beats min^−1^ versus 153.1±4.7 beats min^−1^, *P*<0.05) (Table 1). In contrast, the temperature at which *f*_H,peak_ was reached was significantly different between all groups, with the lowest temperature measured in the rapid screening protocol (20.2±0.4°C) followed by the fish in respirometers (23.2±0.7°C) and, finally, free-swimming fish (26.5±0.4°C) (Table 1). The free-swimming fish also had a significantly higher *f*_H,scope_ (104.2±4.6 beats min^−1^) as compared with the fish in respirometers and fish assessed using the rapid screening protocol (70.8±4.0 and 57.8±3.8 beats min^−1^, respectively) (Table 1). The CT_max_ of free-swimming fish was 27.7±0.4°C, and this value was significantly higher than that of fish tested in the respirometers (25.9±0.6°C) (Table 1, Fig. 1). Both fish in respirometers and free-swimming fish in the tank displayed an increase in activity/movement near their CT_max_.

**Fig. 3.**
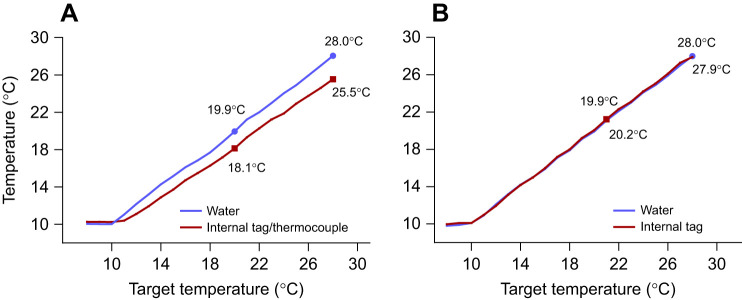
**Comparison of water temperature and internal tag (DST) temperature during the temperature challenge for anaesthetized and free-swimming Atlantic salmon.** The rate of temperature increase was (A) 10°C h^−1^ for anaesthetized fish versus (B) 2°C h^−1^ for free-swimming fish. In three fish, core body temperature was also measured with a calibrated thermocouple. This gave identical data to those recorded by the DST.

The internal (core) body temperature recorded by the DST and the thermocouple was consistently lower than the water temperature during the rapid screening protocol (temperature increase: 10°C h^−1^), and this difference was 2.5°C at 28°C (Fig. 3). In contrast, DST temperature in the free-swimming fish was always consistent with that measured in the water (Fig. 3).

## DISCUSSION

To predict (and implement conservation and management strategies to mitigate) the potential impacts of climate change-related environmental challenges on marine fish populations, it is critical that we have accurate data on their sub-lethal and lethal tolerances. It has been suggested that *f*_H,max_ and its associated rate transition temperature can be used to determine the thermal tolerance of fishes, and that rapid measurements of *f*_H,max_ offer functional and ecological insights into the acute upper thermal limits of this taxon (e.g. their CT_max_) (Ferreira et al., 2014; van der Walt et al., 2021). Thus, the goal of this study was to compare two commonly used methods of determining a fish's *f*_H_ response to acute warming and their CT_max_ with that measured in free-swimming individuals. Further, this study examined the salmon's *f*_H_ response to hypoxia and the oxygen level at which bradycardia was initiated in free-swimming fish versus those post-surgery in a respirometer. Overall, the data reveal that the rapid screening protocol and measuring *f*_H_ responses in a respirometer do not provide values for these important parameters that are quantitatively similar/comparable to those in free-swimming fishes. For example: the ABT as determined by the rapid screening protocol was ∼4°C lower in Atlantic salmon than that determined in a respirometer and in free-swimming fish; the temperature at *f*_H,peak_ differed considerably between the three methods (∼20, ∼23 and ∼26.5°C, respectively); the CT_max_ of free swimming fish was ∼1.8°C higher than that measured for fish in a respirometer fitted with a Doppler^®^ flow probe; and the *f*_H,crit_ and *f*_H_ at 30% air saturation were very different in these two groups. These differences are likely due, in part, to the much higher starting *f*_H_ in the rapid screening protocol and respirometer studies, and the reduced scope for *f*_H_ that this affords. However, the rate of heating/warming, and potential differences between water temperature and the fish's core temperature, must also be considered as potential sources of error/variation.

### Resting *f*_H_

Most commonly, measurements to assess the environmental tolerances of fishes have been performed within a laboratory setting because of the practicality of manipulating conditions in a controlled manner (e.g. changing temperature or dissolved oxygen) and the requirement of being in close proximity to a data acquisition system and computer to monitor physiological variables. More recently, advancements in bio-logging (DSTs) have enabled the continuous monitoring of several parameters in free-swimming fish, with many users recommending a minimum of 1–2 weeks of post-surgical recovery before starting measurements (Bjarnason et al., 2019; Brijs et al., 2018, 2019; Ekström et al., 2018; Hvas et al., 2020). More specifically, studies have noted that while initial stabilization of *f*_H_ takes a minimum of 2–4 days post-surgery, there is a further and more gradual decline in *f*_H_ that lasts for up to 3 weeks (Føre et al., 2021; Hvas et al., 2020; Yousaf et al., 2022; Zrini and Gamperl, 2021). Given the short recovery period of fish fitted with Doppler^®^ flow probes, it is not surprising that the *f*_H_ for salmon at rest was elevated (by ∼20 beats min^−1^, 38%) as compared with fish free-swimming in tanks after 4 weeks of recovery. Assessing the contribution of confinement versus surgery to the reported elevation in the *f*_H_ of fish in the respirometers was beyond the scope of this study. However, Altimiras and Larsen (2000) compared the resting *f*_H_ of rainbow trout (*Oncorhynchus mykiss*) measured in a swim tunnel using biopotentials in the water (i.e. a non-invasive technique) with that of previous studies that used surgical techniques to record this parameter, and reported that *f*_H_ was much lower using the former method. This latter study suggests that stress associated with short post-surgical recovery (18–66 h) was a major contributor to the much higher *f*_H_ in the respirometer-confined salmon in this study. Indeed, Porter et al. (2022) recently measured the plasma cortisol levels of salmon at 24 h post-surgery in a respirometer at 8°C, and these values (mean 47.9 ng ml^−1^) were much greater than those measured in the same population of fish when quickly sampled from their holding tanks (5–10 ng ml^−1^; Vadboncoeur et al., 2023). However, the effects of confinement itself cannot be overlooked. For example, Mignucci et al. (2021) recently reported that gilthead seabream (*Sparus aurata*) implanted with DSTs had a higher *f*_H_ than free-swimming fish (∼75 versus 105 beats min^−1^, respectively) independent of time post-surgery. Clearly, confined and instrumented fish have substantially elevated *f*_H_ and, thus, are questionable to use in estimating resting *f*_H_ in free-swimming fishes in the wild or in aquaculture.

### Hypoxia response

The slowing *f*_H_ in response to decreased/low dissolved oxygen levels, known as hypoxic bradycardia, is an important physiological response and potentially aids in the survival of fish that encounter low levels of oxygen (Farrell, 2007; Stecyk, 2017). This reflex response occurs as a result of increased cholinergic nervous tone within the cardiac branch of the vagus nerve of fishes as described by Stecyk (2017). While the significance of hypoxic bradycardia is not fully understood, several cardiac benefits resulting from this reflex have been proposed, including increased time for diffusion of oxygen into the myocardium, reduced cardiac oxygen demands, increased coronary blood flow during the prolonged diastolic period, and improved cardiac contractility (Farrell, 2007; Joyce et al., 2016; McKenzie et al., 2009).

This response of *f*_H_ to decreasing dissolved oxygen levels has been studied in other salmonids at similar temperatures (10–12°C; e.g. *Oncorhynchus mykiss*; Holeton and Randall, 1967; Marvin and Heath, 1968; Randall and Smith, 1967), and the onset of bradycardia for this species (∼50–65% air saturation) is very similar to what we report for Atlantic salmon in respirometers post-surgery and when free-swimming. Also consistent with this literature is the linear decrease in *f*_H_ reported for salmon in the respirometers. The *f*_H_ for fish in the respirometers decreased by 50% (30 beats min^−1^) from the initiation of bradycardia at 52.6±2.6% to 30% air saturation. The response to hypoxia in free-swimming salmon with DSTs differed in two ways as compared with these fish. First, the *f*_H,crit_ in the latter group occurred at 62.1±2.4% air saturation, at ∼10% higher air saturation than for fish in the respirometers. While there are limited data with which to compare this finding, Mignucci et al. (2021) reported similar results in gilthead seabream (*Sparus aurata*). Mignucci et al. (2021) did not mathematically calculate the breakpoint in the oxygen–*f*_H_ response (*f*_H,crit_); however, based on a visual interpretation of fig. 2 in their paper, it appears that there was also an earlier breakpoint (i.e. decrease in *f*_H_) in free-swimming fish as compared with those in respirometers. Second, the decrease in *f*_H_ between the oxygen level at the initiation of bradycardia (*f*_H,crit_) and 30% air saturation was not linear in the free-swimming salmon. It decreased by ∼24 beats min^−1^ (50%) from 55% to 45% air saturation but did not decrease further. This is unlikely to be a result of the activity level of the free-swimming fish. Although their activity was not quantified in this study, it decreased with the seawater oxygen level, and fish were inactive and just maintaining their position in the water column at the lowest oxygen levels used in this study. This decrease in activity is not a novel finding. For example, Schurmann and Steffensen (1994) reported that Atlantic cod (*Gadus morhua*) decreased their swimming activity threefold between ∼60% and 20% air saturation.

Instead, we propose that this different *f*_H_ response between the two groups may be related to the extent of cholinergic tone on the heart of resting fish in respirometers versus free-swimming fish. Cholinergic tone is a major determinant of *f*_H_ in fish, initiates bradycardia, and has been shown to be affected by several factors (Sandblom and Axelsson, 2011; Wood et al., 1979; Wood and Shelton, 1980). In instrumented Atlantic salmon (confined to a respirometer) at 8°C, cholinergic tone is only 12.4%, and this low tone likely contributed to their higher resting *f*_H_ values (59.8±2.6 beats min^−1^; Porter et al., 2022). It is quite possible that the low *f*_H_ in the free-swimming fish in this experiment was mediated, at least partially, by higher cholinergic tone on the heart and, thus, these fish likely had limited scope to decrease *f*_H_ as the oxygen level in the water decreased. The hypothesis that the reduction in *f*_H_ induced by bradycardia is dependent on resting *f*_H_/cholinergic tone is clearly worth further investigation.

### Acute warming responses

While *f*_H_ increased linearly with temperature in all fish, the ABT was substantially lower for fish exposed to the rapid screening protocol (14.6±0.2°C) in comparison to the ∼18°C measured in both free-swimming fish and those in respirometers. This is a large (and important) difference, and would have been even greater if, as for most previous studies using the Casselman et al. (2012) protocol, we had only measured water temperature. In this study data loggers simultaneously recorded *f*_H_ and internal (core) temperature during the rapid screening protocol, and this temperature differed from that of the water (Fig. 3), whereas this difference was not seen in the free-swimming fish at a warming rate of 2°C h^−1^. While the size of the salmon exposed to the rapid screening protocol (∼800 g) likely contributed to the large difference in core versus water temperature that we report, this overall result/finding is not surprising. A difference of 1–2°C has been reported for small Atlantic cod (*Gadus morhua*; ∼100 g) and a 0.2°C discrepancy has even been reported in zebrafish (*Danio rerio*) (Jutfelt et al., 2019; Morgan et al., 2019). In addition, Sandblom et al. (2016) identified this issue in preliminary studies with perch (*Perca flaviatilis* L.) and reduced the rate of heating in their rapid screening protocol to 3°C h^−1^ to avoid this issue. These above issues question the use of the rapid screening protocol (especially at high rates of heating) for determining the ABT of *f*_H_ and, thus, its accuracy when estimating the *T*_opt_ of fishes.

**Fig. 4.**
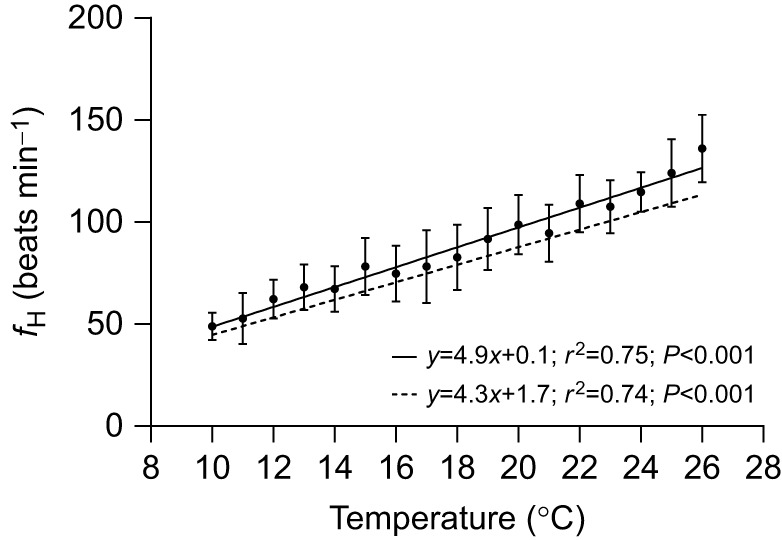
**Comparison of the average *f*_H_ of free-swimming Atlantic salmon implanted with DSTs during a temperature challenge at 2°C** **h^−1^.** The solid line represents the linear regression for this relationship in fish (*n*=12) in this study (with the symbols indicating means±s.e.m.). The dashed line is the daytime linear regression reported in Gamperl et al. (2021) for free-swimming fish (*n*=5) in a commercial sea-cage implanted with *f*_H_ DSTs during a summer heat wave in Newfoundland in 2019.

With respect to estimating/determining maximum cardiac values related to upper thermal tolerance, the rapid screening protocol also gives very different values when compared with those of free-swimming fish implanted with DSTs and given a long (appropriate) period of surgical recovery. In fact, *T_f_*_H,peak_ was >6°C lower with the rapid screening protocol (20.15±0.4 versus 26.49±0.4°C), and this value was ∼7.5°C lower than the CT_max_ of the free-swimming fish implanted with DSTs. This is the first study to directly compare these methods for determining thermal tolerance-related parameters in fishes and raises significant concerns about the use of this protocol for determining peak *f*_H_ values (and thus upper temperature tolerance) in fishes, and the suitability of using values derived from this method for predicting the impact of marine heatwaves on the distribution and survival of fishes. This is particularly true given the confidence we have in the temperature-related data we obtained in the free-swimming fish. The temperature–*f*_H_ relationship obtained for these fish is extremely similar to that reported by Gamperl et al. (2021) for salmon implanted with DSTs in a commercial sea-cage during a marine heat wave (see Fig. 4). In comparison to the rapid screening protocol and the free-swimming fish with DSTs, the data for fish in the respirometers was intermediate. The ABT and *f*_H_ were comparable to values for the free-swimming fish, whereas *T_f_*_H,peak_ was ∼3.5°C lower.

**Fig. 5.**
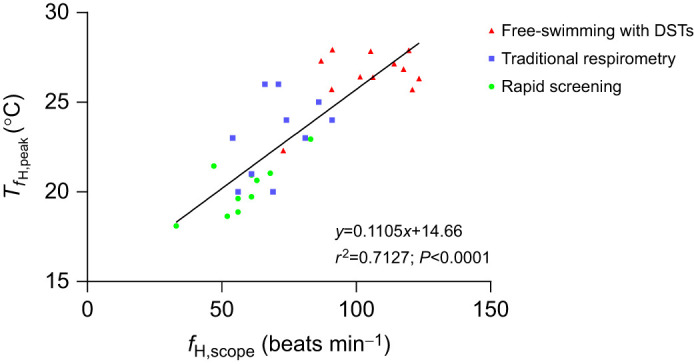
**Relationship between the scope for heart rate (*f*_H,scope_) and the temperature of peak *f*_H_ (*T_f_*_H,peak_).** The symbols represent each treatment group: circles, fish anaesthetized and implanted with DSTs (*n*=11); squares, fish implanted with Doppler^®^ flow probes and tested using traditional respirometry (*n*=10); and triangles, free-swimming fish held with conspecifics and implanted with DSTs (*n*=12).

With regard to what is potentially mediating the difference in *f*_H,peak_ (and thus the estimates of upper thermal tolerance that would be derived from them) between these experiments, the data are quite revealing. In the rapid screening protocol, resting *f*_H_ following injections of atropine and isoproterenol was artificially elevated (69.9±1.1 beats min^−1^), and this in addition to a lower *f*_H,peak_ (126±4.5 beats min^−1^) limited the fish's *f*_H,scope_ (57.8±3.8 beats min^−1^) as compared with free-swimming conspecifics with DSTs (104.2±4.6 beats min^−1^). Indeed, if you plot the *T_f_*_H,peak_ for all three groups versus their *f*_H,scope_ (Fig. 5), there is a very strong positive relationship (*r*^2^=0.713) between these two parameters. Collectively, these data highlight the importance of the available scope for *f*_H_ in determining a fish's upper thermal tolerance, and of eliminating or reducing stress (and therefore resting *f*_H_) in fish used in protocols designed to determine their upper thermal tolerance. Further, they emphasize how stressors in the natural environment or in aquaculture operations could result in these animals being more susceptible to acute increases in temperature.

We were not able to measure CT_max_ for fish given the rapid screening protocol as the salmon were anaesthetized throughout, or confidently determine their *T*_arr_ (the temperature at which arrhythmias first occur) from the ECG recordings in fish with DSTs as the measurement period in the tags used was only 6 s. However, we did obtain values for *T_f_*_H,peak_ and CT_max_ of the free-swimming salmon and those tested in respirometers. Despite showing no significant difference in *f*_H,peak_, the free-swimming fish had values for *T_f_*_H,peak_ and CT_max_ that were ∼3.3 and 1.8°C higher, respectively, than those of fish in the respirometers. That the CT_max_ of these two groups was quite similar/comparable is not surprising. Based on a number of studies that have compared CT_max_ between different fish populations/families (Anttila et al., 2013; Bartlett et al., 2022; Ignatz et al., 2023), those acclimated to temperature differences of <10°C (Beitinger et al., 2000; Anttila et al., 2015; Morgan et al., 2019), or exposed to suboptimal conditions (e.g. hypoxia; Anttila et al., 2015; Ern et al., 2016), it appears that CT_max_ displays limited plasticity/variability (∼1.5–3.0°C) when values are compared at the same rate of heating. This is likely due the specific mechanisms that determine when a fish loses equilibrium (this parameter defining when a fish has reached its CT_max_) (Ern et al., 2023).

### Summary and perspectives

There is accumulating evidence that the geographical distribution of aquatic ectotherms can be predicted based on their thermal limits (Sunday et al., 2012; Stuart-Smith et al., 2017; Payne et al., 2021), and that increases in the frequency and severity of temperature extremes (i.e. heat waves and ‘cold shocks’/‘cold stress’) will be important determinants of the survival and population strength (biomass) of fish species in the future (Cheung and Frölicher, 2020; Cheung et al., 2021; Genin et al., 2020; Perry et al., 2005; Szekeres et al., 2016; Reid et al., 2022). Thus, obtaining meaningful and accurate values for the thermal limits of various species-specific biological processes will be key to implementing conservation and management strategies (McKenzie et al., 2016; Ørsted et al., 2022; Desforges et al., 2023; Bates and Morley, 2020). This may be particularly true with regard to the impacts of heat waves as recent analyses suggest that the heat failure (mortality) rate of ectothermic species, including fishes, doubles (on average) for every 1°C increase within the stressful temperature range (Jørgensen et al., 2022; Ørsted et al., 2022). While CT_max_ (loss of equilibrium) has been used by a number of authors to estimate the upper thermal tolerance of fishes, the rates of heating used are often faster than ecologically relevant (i.e. >5°C h^−1^; Caissie et al., 2012; Desforges et al., 2023; Richards, 2011; Rodnick et al., 2004; Gilbert and Farrell, 2021), and others have suggested that CT_max_ is not the most ecologically relevant measure of a fish's thermal tolerance (Bartlett et al., 2022; Ignatz et al., 2023). For example, high rates of heating do not allow for the balance in damaging and regenerative (or ‘plastic’) responses that ultimately define critical boundary temperatures (Ørsted et al., 2022). Thus, other protocols and methodologies need to be developed, and evaluated/validated, with regard to determining the thermal tolerance of fish to short-term (acute) warming events.

In this first of its kind study, we compared the temperature-dependent *f*_H_ responses of Atlantic salmon when exposed to temperatures approaching their upper thermal limit using three different protocols/experimental approaches. This study clearly shows that there are major differences in temperature-dependent *f*_H_ parameters obtained using the three methods, and that the rapid screening protocol greatly underestimated the thermal limits/tolerance of free-swimming Atlantic salmon. This finding is troubling given the number of researchers using this protocol to assess the upper thermal tolerance of fishes. Further, it highlights the rapid screening protocol's limitations with respect to determining the upper thermal tolerance of fish in their natural environment and suggests that this protocol should not be used to predict the vulnerability of wild fishes to heat waves (e.g. van der Walt et al., 2021). However, it is not surprising as this protocol (Casselman et al., 2012) was originally designed to examine whether ABT could be used to estimate a species *T*_opt_ for aerobic scope, not a species’ upper thermal tolerance. Also, the injection of atropine and isoproterenol artificially elevates the fish's initial (resting) *f*_H_ and, thus, reduces the available scope to increase *f*_H_ – a parameter which our results suggest may be a key factor in determining a fish's environmental tolerances/limits (e.g. see Fig. 5).

Instead, our results support the conclusion of Mignucci et al. (2021) that biologging on free-swimming fishes provides reliable, ecologically relevant, insights into the cardiac and behavioural responses of fish to environmental stressors and suggest that the *T_f_*_H,peak_ of free-swimming fishes is a good/accurate predictor of the upper thermal tolerance of this taxa. This is based on the extremely similar relationship between *f*_H_ and temperatures from 10 to 20°C in this lab-based study and that determined for Atlantic salmon in a large sea-cage recorded during a heat wave in Newfoundland (Gamperl et al., 2021; Fig. 4), and that the *T_f_*_H,peak_ and CT_max_ of salmon in this study were within 1.8°C. In addition, there are several features of DSTs that make them a valuable tool for assessing fish thermal biology going forward. First, the cost of heart rate DSTs is decreasing, and there are DSTs that are capable of simultaneously recording *f*_H_ and activity (3D acceleration). These aspects will allow DSTs to be used by a broader range of scientists, and to account for changes in behaviour (e.g. swimming speed: Zrini and Gamperl, 2021; Warren-Myers et al., 2023) when using them to examine aspects of the thermal biology and bioenergetics of fishes. There are also DSTs now available that are capable of recording ECGs for intervals up to 15 s at 100 Hz (as opposed to the 6 s recording limit in the DSTs used in this study) which will make the accurate determination of *T*_arr_ (although we believe *T_f_*_H,max_ is a more ecologically relevant measure of a fish's thermal tolerance) possible. Finally, the environmental tolerances of several individuals can be measured simultaneously. The latter point is important as this allows for data on a large number of fish to be collected in a relatively short period of time, and this was one of the arguments for developing the rapid screening protocol.

This, however, is not to say that there are no drawbacks to using this experimental approach. First, DSTs have a limited battery life, they cannot presently be implanted into very small fish, and measuring *f*_H_ in free-swimming fishes requires that you have an appropriate place to hold the fish post-implantation. Second, measuring *f*_H_ in free-swimming fishes does not allow one to examine the mechanistic basis/es for differences in environment-dependent *f*_H_ responses and tolerances between species, populations or as impacted by specific conditions. In this regard, our results suggest that protocols used to measure heart function in fishes, and that allow for the measurement of other physiological parameters (e.g. oxygen consumption) and blood sampling/agent administration, could be modified so that they provide more accurate measures of thermal tolerance. The salmon implanted with Doppler^®^ flow probes had equivalent values for the ABT for *f*_H_, a value for *f*_H,peak_ that was not significantly different from that of free-swimming fishes, and a CT_max_ that was only 1.8°C lower as compared with the latter group. It is possible that longer surgical recovery times, and approaches to lower confinement stress (e.g. Altimiras and Larsen, 2000), could lower the fish's resting *f*_H_ and, thus, allow the fish to have a more realistic *f*_H_ scope available to meet environmental and other challenges.

Finally, our results highlight the potential interaction between the degree of stress experienced by fishes, and how this influences their capacity to respond to other environmental challenges. With regards to *f*_H_, it has already been shown in sablefish (*Anoplopoma fimbria*) (Leeuwis et al., 2021) and Atlantic salmon (A.K.G., J. J. H. Nati, K. A. Clow, R.M.S., L. Gerber, E. F. C. Peroni and E. S. Porter, unpublished data) that the cholinergic-mediated bradycardia induced by hypoxia prevents fish from being able to increase *f*_H_ during an acute thermal challenge and reduces their thermal tolerance (CT_max_). In addition, in this study, we suggest that: the low *f*_H_ in truly resting (unstressed) fish limits their capacity to reduce *f*_H_ when exposed to oxygen-limited conditions (i.e. due to an already high cholinergic tone on the heart); and that stress-induced increases in *f*_H_ limit the *f*_H,scope_ available to deal with rising temperatures and, thus, result in a reduction in upper thermal tolerance. Both of these observations/hypotheses require validation via additional experiments, but potentially add greatly to our understanding of fish cardiorespiratory physiology and the mechanistic basis of environmental tolerances.

## Supplementary Material

10.1242/jexbio.246227_sup1 Supplementary information
