# Supplementary material for: The upper temperature and hypoxia limits of Atlantic salmon (Salmo salar) depend greatly on the method utilized
Source: J Exp Biol. 2023 Sep 26;226(18):jeb246227. doi: 10.1242/jeb.246227 (PMC10560559; doi:10.1242/jeb.246227)
Supplement: Supplementary information [file jexbio-226-246227-s1.pdf]

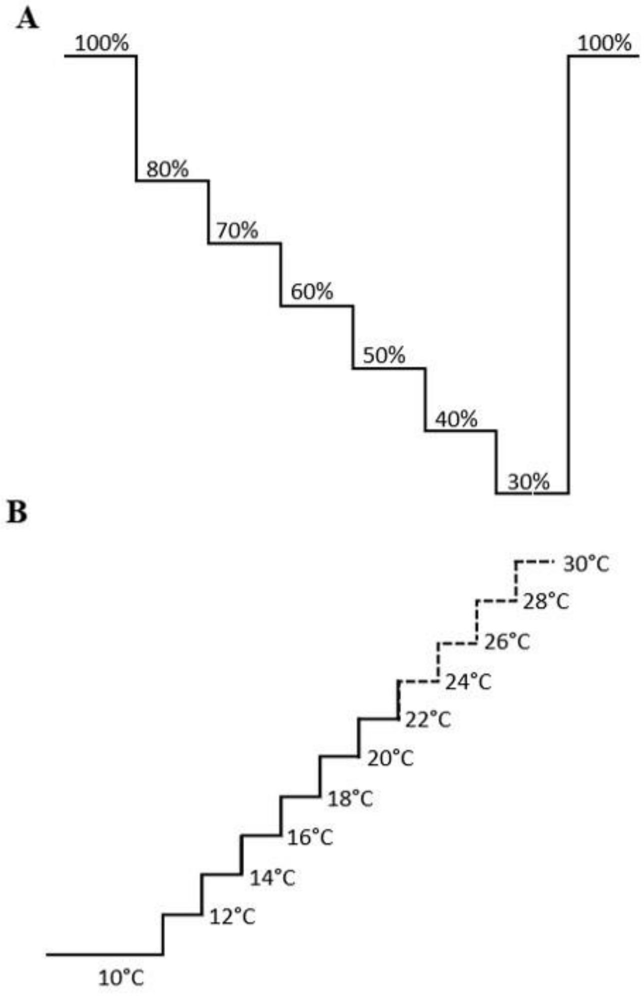

Fig. S1. Schematic representation of changes in water oxygen level (% saturation) and temperature (°C) during the hypoxia (A) and temperature challenges (B), respectively. These protocols were used for both fish tested using traditional respirometry and for free-swimming fish. Each step represents 1 hour increment. Fish tested using the 'rapid screening protocol' underwent a similar temperature increase at 10°C h $^{-1}$.

Journal of Experimental Biology • Supplementary information

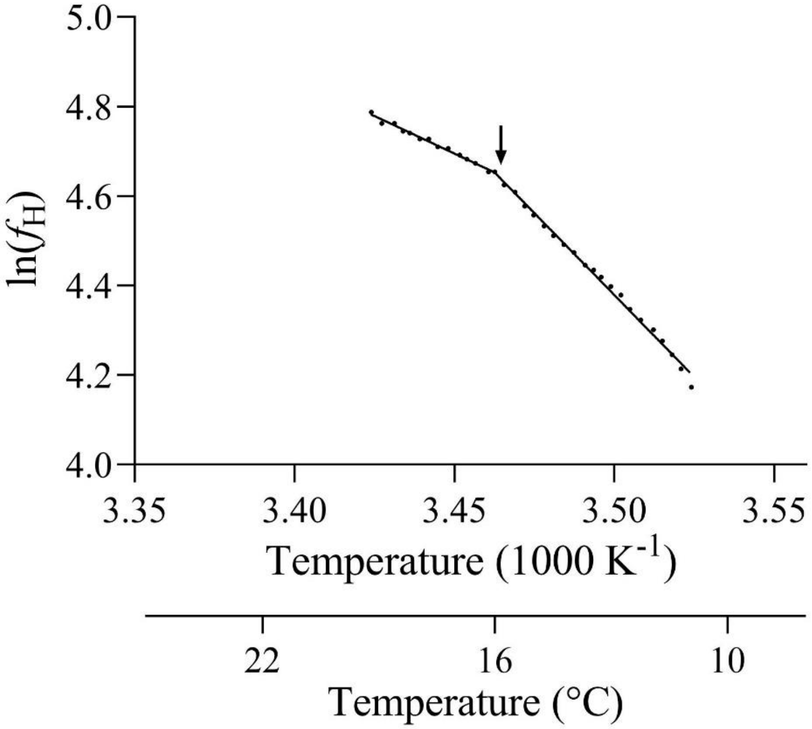

Fig. S2. Arrhenius breakpoint plot of the natural log of the maximum heart rate (ln f_{HMax}) against the body temperature, plotted as the inverse of temperature in Kelvin (K). Secondary x-axis notes temperature in Celsius (°C) for reference. The point at which the change in slope occurs is the Arrhenius breakpoint temperature (ABT) indicated by (↓).

Table S1. Statistical summary of values for ANOVAs, unpaired t-tests and linear regressions. **(I)** Fish anesthetized and implanted with DSTs (n=11), (**II**) fish tested using traditional respirometry and implanted with Doppler® flow probes (n=10) and (**III**) free-swimming fish held with conspecifics and implanted with DSTs (n=12). Bolded *P*-values are statistically significant (*P* > 0.05).

		F	R^2	*P*	N1	N2
ANOVA and Tukey's Post Hoc Summary						
Mass vs. Group		1.180	0.07	0.3212		
Length vs. Group		1.033	0.06	0.3683		
RVM vs. Group		4.244	0.2205	**0.0238**		
	I vs. II			**0.0251**	11	10
	I vs. III			0.0941	11	12
	II vs. III			0.7554	10	12
ABT vs. Group		4.641	0.2967	**0.0208**		
	I vs. II			0.0592	11	7
	I vs. III			**0.0384**	11	7

Journal of Experimental Biology • Supplementary information

	II vs. III			0.9807	7	7
f_{Hrest} vs. Group		53.56	0.7812	**<0.0001**		
	I vs. II			0.8508	11	10
	I vs. III			**<0.0001**	11	12
	II vs. III			**<0.0001**	10	12
f_{Hpeak} vs. Group		8.867	0.3715	**0.0009**		
	I vs. II			0.1753	11	10
	I vs. III			**0.0006**	11	12
	II vs. III			0.0837	10	12
Tf_{Hpeak} vs. Group		37.86	0.7162	**<0.0001**		
	I vs. II			**0.0011**	11	10
	I vs. III			**<0.0001**	11	12
	II vs. III			**0.0004**	10	12

Journal of Experimental Biology • Supplementary information

f_{Hscope} vs. Group		34.52	0.6971	**<0.0001**		
	I vs. II			0.0940	11	10
	I vs. III			**<0.0001**	11	12
	II vs. III			**<0.0001**	11	12
$Q_{10PreABT}$ vs. Group		7.744	0.4131	**0.0028**		
	I vs. II			**0.0020**	11	7
	I vs. III			0.1792	11	7
	II vs. III			0.1685	7	7
Unpaired T-Test						
CT_{Max}	II vs. III	29.76	0.3349	**0.048**	10	12
f_{Hcrit}	II vs. III	1.436	0.2926	**0.0113**	9	12
f_H 100%	II vs. III	2.855	0.8162	**<0.0001**	10	12
f_H 30%	II vs. III	1.058	0.4389	**0.0011**	10	11

Journal of Experimental Biology • Supplementary information

Linear Regression					
f_{Hscope} vs T_{fHpeak}		79.38	0.7127	**<0.0001**	34
f_H vs Temperature	III	609.2	0.7510	**<0.0001**	204
